# The Physiological Therapeutics of Reasoning Madness

**Published:** 1900-01

**Authors:** C. A. F. Lindorme

**Affiliations:** Chicago, Ill.


ATLANTA

### Journal-Record of Medicine.

Successor to Atlanta Medical and Surgical Journal, Established 1855, and Southern Medical Record, Established 1870.

VOL. I. JANUARY, 1900. NO. 10.

EDITORS { BERNARD WOLFF, M.D., DUNBAR ROY, M.D. — M. B. HUTCHINS, M.D., BUSINESS MANAGER.

PUBLISHED MONTHLY. OFFICE 305 AND 306 FITTEN BUILDING.

### *ORIGINAL COMMUNICATIONS.*

### THE PHYSIOLOGICAL THERAPEUTICS OF REASONING MADNESS.

BY C. A. F. LINDORME, PH.D., M.D.,

CHICAGO, ILL.

It has been claimed, and we believe with good reason, that physiology, more than other branches of medicine, can be called a science. This implies, however, that physiological therapeutics must participate in such qualification, and this points out the road we have to follow in this investigation in order to understand reasoning madness. It is indispensable to know, first, exactly what is reason, the function of the sane intellect. The conundrum of a concomitancy of the most pronounced madness with the finest culture of the intellect, be it in a Nietzsche or a Don Quixote, in a Tasso or Hamlet the Dane, was the puzzle of the past centuries, and it is of our own. One might accuse our time of retrogression, even, insomuch as it has added to the intricacies by pointing out the neutral field between the dimness of the extremes, the appallingly extending borderland of insanity, if the reader, in well-timed perspicacity, does not prefer to augur from the new complication the survening of a solution.

Routine physiology holds that all mental function is restricted to the action of the nerves, the spinal cord, and the brain. Positive demonstration, however, was never accomplished. "Just as it is the special function of the muscle-fibre to change its form when it is excited, so it is the special function of the nerve-fibre to transmit the condition or activity excited at one end throughout its length, and to awaken the activy of the cell with which it communicates. Nerve-fibres are the paths of communication between nerve-cells in the central nervous system, between sense-organs at the surface of the body and the nerve-cells, and between the nerve-cells and the muscle- and gland-cells. Nerve-fibres are distinguished as afferent and efferent, or centripetal and centrifugal, according as they carry impulses from the surface of the body inward, or from the central nervous system outward." (Warren P. Lombard in Am. Text-book of Phys.) It is added: "By an irritant is meant an external influence which, when applied to living protoplasm, as of a nerve or a muscle, excites its action." Now, the American Text-book, by authors and publishers, was intended to convey the ruling doctrine of science, not any opinion built up with results only of merely subjective speculation. We may use it, therefore, as safe reference, and hence the principle must be established, and is established by science, whether explicitly enunciated or not, that the virtue of a nerve is decidedly a *passive* virtue. Be it ever so true that the faculty of understanding is in the nerves, it is none the less a verity that this faculty is not an independent nor a spontaneous one, but one which acts only on the provocation of an excitant.

This fact wants watching by the physiological therapeutist, for reason of the bearings on the clinical circumstances.

"As far as has been ascertained, the nerve impulse has the same general characteristics in all forms of nerves, medullated and non-medullated, sensory, inhibitory and motor, and, except as regards strength, rhythm, etc., is the same whether they be excited artificially or normally by a nerve-cell or sensory end-organ." (Lombard, 1 c.) Furthermore, physiology states that the nerve-fibres run their course, separately, when being aggregated, even, in bundles, and from this we must conclude that, at the end-station, every nerve-message is confined to the contents it was given at the starting point.

Accordingly, if even from the nature of a nerve as devoted to the understanding, or a function which must as a matter of course be brought under way by impressions of the things to be understood, its passiveness had not to be concluded, the accepted axioms of physiolgy would impel us to argue that way, and consequently the student of mental alienation remain at sea about the freaks of intellection we meet with according to the supposed degrees of impaired health. Since in the nerves the single impressions only are registered which at their starting point they received, where is in the brain the occasion for the comparing, sifting and ulterior intellectualizing of the single data extant? It is a notorious fact that in the single data conveyed by the nerves to the brain there is so little of the knowledge we possess of the world, all the evidence of these data being the source of the same notwithstanding, that it would be impossible even to neurology to analyze in the said knowledge the constituents of its composition.

The neurologists, in the deficiency of their nerve-theory, try to get under cover of the nerve-cell, as the comprehensive element. But physiology teaches that the functions of the nerve-fibre is "to awaken the action of the cell with which it communicates." (Lombard, 1 c.) As to a general intellection in the nervous system we are left accordingly. There is no corresponding collective action.

Thus, even, if physiology were to give us no more reason for seeking beyond the nervous system for an explanation of the psychological problem, we should find it in the circumstance that the latter cannot be explained through mere nerve-action. But physiology admits this directly herself, and this unanimously: "The vascular system is a path of communication between the several organs and the tissues, and the circulating blood is a medium of exchange. The blood carries nutritive material from the digestive organs and oxygen from the lungs to all the tissues of the body, and it transports the waste materials which the cells give off to the excretory organs. In addition to these functions it has the power to neutralize the acids which the cells produce during action, and so maintains the alkalinity essential to the life of the cell; it supplies all parts with moisture; by virtue of the salts which it contains, it insures the imbibition-relations which are necessary to the preservation of the normal chemical constitution of the cell-protoplasm; it distributes the heat and so equalizes the temperature of the body; finally, in addition to these and similar other functions, it is itself the seat of important chemical changes, in which the living cells which it contains play an active part. It is not strange that such a fluid should exercise a marked influence upon the irritability of the nerves and muscles." (Lombard, 1 c.)

"Of the substances supplied to the muscle by the blood, oxygen is one of those the want of which is soonest felt. (*Id.*)

"The nerve-fibre demands a constant supply of blood for the maintenance of its irritability. The irritability of the nerve cannot continue long without oxygen." (*Id.*)

"When the muscle is stimulated to action, its blood-vessels are at the same time dilated so that it receives free supply of blood. (*Id.*)

"The finest network of vessels is, however, to be found where the cell-bodies are most densely congregated, and indeed the distinction between the masses of gray and white matter in the central system is as clearly marked by the relative closeness of the capillary network as in any other way. One result of this relation between the blood-supply and the cell-bodies which form the gray matter is a general arrangement of the vessels along the radii of the larger subdivisions of the brain, as the cerebral hemispheres and the cerebellum." (Henry H. Donaldson, 1 c.)

From all this it follows that, whatsoever function be ascribed to the nerve-cell, it is owing only to the blood action in it that such function can be sustained. It is a mistake, therefore, of the older theory to give credit to the nerve-cell for its origination of the field of mental action, without taking into account the indispensable concomitancy of the capillary. It was done on the authority of an experiment of Vogt and Moleschott. But this experiment was unscientific. In an experiment the effect attributed to a cause must be *isolated*. In the extirpation of the brain, however, as done by Vogt and Molsechott, which was claimed to prove the nerve-matter of the brain as the only indispensable instrumentality of the mind, such isolation was entirely neglected, would moreover have been technically altogether impossible, and this invalidates the whole former standpoint as completely unfit to base a psychiatric theory on.

In the more recent years much attention has been given to the histology of the blood. But the physiological properties were noted without the slightest suspicion of a psychological bearing of the same. The investigators gave lengthy descriptions of the red corpuscles, of their disklike shape, exhibited in rolls, and mentioned as particularly characteristic of the scientific framework of their structure which insures an individuality to every one of them, and imparts to them a peculiar power of refraction which, from the utilization without which nature never is found, it must be assumed, does some service. But that this service is psychological they never hinted. Physiologically the red blood-corpuscles are subservient to the conveyance of oxygen. But the chemical requirements connected therewith do certainly not carry with them the necessity of a disklike shape and a peculiar tissue frame, evidently destined for individual performance.

The representation of the material world is accomplished in the nervous system. But the personal realization is reserved to the red blood-corpuscles; the material world is not recognized except according as the red blood-corpuscles do it, and they do it to suit themselves. The perception of the outer world through the nerves is an objective process; it is in strict keeping with the cosmetic rule of cause and effect. But this relation does not extend to the red blood-corpuscles; owing to the floating condition of the latter the nerve impressions do not work unconditionally as a cause, but leave the blood-corpuscles in free subjectivity of a choice; the physicial condition of the nerve-cells, on the one hand, of the disks of the blood on the other hand, represent the material world with its rigid ways, but the relativity of both, in their joint action, is the mentality in which is exhibited the free independent personality, or the SOUL of man.

There is nothing in all physiology to array against this, while psychiatry becomes explicable first through our theory. With the blood as determining factor, there is nothing surprising any more in the abiding of the most indubitable intellectual competence fairly, with the most stupendous mental alienation. Nor is it surprising that in the borderland of insanity it is not among the inferior heads that we find the cranks. The crankiness is not found in the analysis of the nervous impressions; it is found in the synthesis of the reception in the blood-disks. And as there is nothing which helps more to see a thing than to see it grow, we will have an example from the domain of evolutionary intellection: Audubon, one evening at the Ohio river, noticed a number of blackbirds taking their flight across the water overtaken by a goshawk. The number was large enough for a sport, but did not promise to remain so for long, because once past the river there was in the dense forest promising chance of shelter. Following the crude habit of birds of his class, the hawk would have devoured the birds as soon as caught one by one, and would not have got half enough before losing his chance. What did Audubon see him do to improve the occasion? He saw him drop deliberately each blackbird killed, and he kept on killing and dropping as long as the free field gave an opportunity, and then one by one collected from the water's surface the dead bodies which on it were floating, carried them singly to a convenient place and made his supper.

Now, the question is, what inference have we to make from the above occurrence, which, being reported by Audubon, is undoubtedly true, with regard to the mentality involved? There are professors who would give the opinion that there is an inkling of mechano-mathematical science in it, and there is no telling what an undergraduate in zoology would try to prove in a dissertation on the subject. Nevertheless there is in the stratagem not a greater exhibit of intellectuality than in the routine-practice of a dog or a cat in grappling with an adversary, to jump at his throat, or of little ducks, wanting flies and swimming insects, to navigate on the neighboring pools, all the warning shrieks of the incubating hen-nurse notwithstanding. There was an exhibit of keener sense than is usually observed. But this sense was as animalistic as appetite could make it; it resulted directly from the hawk's desire to eat, and not only to eat at all, but to eat his fill. So far from justifying us to conclude superior intellectuality, a higher degree of encephalic evolution, we may infer from what the goshawk did no more than individually strongly developed hawkish qualities; he did not prove remarkable on account of the data he scored in his brain, but on account of the sportsmanship he displayed in making use of his experience for the benefit of his animalistic purposes.

The attentive reader will find no difficulty in admitting what we have said on the score of the goshawk. But there will be many who will hesitate to accept the similar category in man. But the rule that the nervous system stores the data of observation, the utilization of which is determined according to the finding of the red blood-corpuscles, the data in the nerve-cell working as motives of action in the blood, is precisely the same hither and thither. And as usual, it is in pathological cases that most drastically is confirmed the physiological relation. So, Tuke reports that an asylum physician who had an inmate who believed he had discovered the *perpetuum mobile*, decided to carry him to Arago, when this eminent scientist had them visit Alexander von Humboldt, thinking that such twofold overweening authority would shame the lunatic into reasonability. Well, they went, and apparently with entire success. The arguments of the illustrious men carried all before them in so far as intellectuality was concerned. But the rub was that this was not at all the point at issue, and no sooner the lunacy practitioner with his case was in the street again, then the lunatic exclaimed: "And yet Mr. Arago is wrong; I am right." The thing was, the hitch was in the blood, not in the brain.

We will not enter here deeply into the discussion of the question whether there is such a thing as pure intellectuality, but need hesitate so much less to concede the point, as it would by no means militate against our theory. If there is such a thing as pure intellectuality, it can be only by virtue of the purity of the intention in which the intellectuality is made use of, and this, according to the testimony of physiology, is realized in the *drift* of the red corpuscles from which such intention derives. We may from our standpoint emphasize the position even. The culmination of true intellectuality must be truth. Now, then, truth was never attained except by those who were prompted by the love of truth more than by meaner interests. That which ruled was the will for all that.

The inferences which must be made from the above with reference to the physiological therapeutics of reasoning madness are of very great importance. For, although it follows from our demonstration that the service done by the nervous system is restricted to the scoring of the data of sense-action, and that consequently the coloring which is hand and glove with all sifting and grouping, comparing and concluding, is perpetuated in the blood, or, more specifically, in the red corpuscles of the same, yet it is a recognized fact that it is worked upon the latter through the former. In the first instance it is done by HABIT: As long as the individual is young, before becoming aware of any great prominence of inclinations, it runs with the crowd, in narrow or wider environment, family, community, state, nation. That is the period which wily educators have always chosen for the inculcation of bad seed. It has hardly ever been used for the propagation of noble sentiments, but can with advantage be made avail of in the interest of virtue as well as any vicious quitch for which the germs are laid in those days of tender turning. And, aside of the general rules which apply in all directions, there is, with regard to insanity, no habit more remedially effective than that of CRITICAL THOUGHT. It is a notorious fact, that among the inmates of lunatic asylums, the *scholars* have the lowest pregnancy figure. If it is the will in the red blood-corpuscles which is sovereign, yet it is the nervous system in which the will has to look for his material, and as a prudent manager raises good workmen, so a well-trained will raises good thought, above all *the habit of correct thought.* This, however, is the sentiment from which virtue grows; it is the scorning of life; and this carries with it candidness and all the positive good-will of which nature, naturally, is so full. For, it is altogether erroneous to imagine nature congenitally bad. Psychopathic conditions are not necessarily preternatural. But they are by far more the result of misunderstood than of vicious will. And, once the habit settling into society to promote the good, there is by far more chance of success in this than in its contrary.

To close with, we will mention the notable circumstance that therapeutical psychiatry proceeded practically on this line, although theoretically deep in metaphysics yet. In the asylums it is the general practice now to make use of the occupations which lead back to the more simple modes of thought, as agriculture, gardening, artisanship, and the like. But it should not be forgotten that in its roots the same psychology obtains in all humanity, not mentioning, as more specifically analogous, the wide territory of the borderland of insanity. Thus, what an outlook, if the same beneficent physiological therapeutics could be applied to all mankind?

