# Errata

**Published:** 1900-02

**Authors:** 


### ERRATA.

In my entire literary career the printer's devil has never played me a meaner trick than in the quandary below. One consolation I found, however, in my exasperation: This was a confirmation of my psychological theory: If the typesetter, in reading the manuscript, had gone by the data of observation, as per eyesight, merely, all would have come out right. But his will tampered with the result; he wanted to show how the world would be if he had had to see in the making; hence the monstrous abomination of the exhibit:

P. 725, line 7 from below, instead of "cosmetic" read cosmic.

P. 727, line 13 from below, instead of "them," etc., read the visit of.

P. 728, line 5 from above, instead of "true" read pure.

P. 728, line 15 from above, instead of "perpetuated" read perpetrated.

P. 728, line 8 from below, instead of "pregnancy" read frequency.

P. 728, line 2 from below, istead of "scorning" read conforming.

LINDORME.

